# Interpretable Machine Learning for Identifying Key Variables Influencing Gold Recovery and Grade

**DOI:** 10.3390/ma18184318

**Published:** 2025-09-15

**Authors:** Sheila Devasahayam

**Affiliations:** Western Australian School of Mines: Minerals, Energy and Chemical Engineering, Curtin University, Kalgoorlie, WA 6430, Australia; sheila.devasahayam@curtin.edu.au

**Keywords:** gold flotation, explainable ML, SHAP, feature importance, recovery, grade

## Abstract

Gold flotation performance is influenced by multiple interacting variables, yet most predictive studies in this area emphasize accuracy while neglecting interpretability, limiting their practical value for process engineers. This study applies explainable machine learning techniques to identify and interpret key variables, controlling cumulative gold recovery and grade using a small, experimentally derived dataset (n = 11) from Ballarat gold ore flotation. A Gradient Boosting Regressor, combined with SHAP (Shapley Additive Explanations), permutation importance, and feature importance analyses, was employed to uncover both linear and non-linear relationships. Power, head grade, and processing time consistently emerged as dominant predictors, while interaction effects (e.g., head grade × collector, size × head grade) provided additional explanatory insights. The findings reveal actionable process implications, including trade-offs between energy input and flotation efficiency, and highlight operational conditions for improved recovery and grade. This study demonstrates that interpretable machine learning can bridge the gap between statistical modeling and process optimization, delivering transparent, domain-specific insights even in data-constrained environments.

## 1. Introduction

Flotation remains a critical separation technique for extracting valuable minerals from ores, where operational parameters such as grinding Power, particle size, head grade, and reagent type strongly influence both recovery and product grade [1,2,3]. The inherent complexity of flotation systems, driven by non-linear interactions between physical, chemical, and operational variables, presents significant challenges for process optimization [4,5]. Traditionally, empirical models or response surface methodologies have been employed to identify key factors; however, these approaches often fail to capture intricate feature interactions and non-linear dependencies that govern flotation performance [6,7].

In recent years, machine learning (ML) has emerged as a Powerful tool for analyzing complex metallurgical processes [4,8,9,10,11]. Applications in mineral processing have included predictive models for recovery, reagent optimization, and energy efficiency using algorithms such as Random Forests, Support Vector Machines (SVM), and Neural Networks [12,13,14,15]. While these models achieve high predictive accuracy, they are frequently criticized for being black-box approaches that offer little interpretability, limiting their adoption in industrial settings where explainability and domain-driven decision-making are essential [16,17].

To address this limitation, explainable artificial intelligence (XAI) methods such as SHapley Additive exPlanations (SHAP) have gained traction in other sectors, including energy systems, healthcare, and manufacturing [18,19,20]. SHAP offers both global and local interpretability by quantifying the contribution of each feature to individual predictions and the overall model outcome [21]. However, its application in mineral processing, especially for interpreting flotation performance, remains scarce. Most existing work in flotation modeling focuses on predictive accuracy rather than understanding feature importance and interactions in a physically meaningful context [22].

This study departs from the predictive modeling paradigm and focuses on interpretability. Specifically, it applies Gradient Boosting Regressor combined with SHAP, permutation importance, and feature importance techniques to identify key variables and interactions influencing cumulative gold recovery and grade. The dataset used is experimentally derived and intentionally small (n = 11), reflecting practical constraints often encountered in mineral processing plants, where large-scale datasets are difficult to obtain. Rather than being a limitation, this scenario underscores the need for interpretable methods capable of extracting actionable insights from limited data [23].

The novelty of this work lies in

Introducing interpretable ML techniques (SHAP, permutation importance) to flotation analysis for revealing global and local feature contributions, non-linear effects, and interactions.

Demonstrating the feasibility of SHAP-driven insights in data-constrained environments, a common challenge in mineral processing operations.

Linking model interpretability to operational decision-making, such as energy optimization, particle size control, and reagent selection.

Based on these objectives, the study addresses the following research questions:

Which operational variables most strongly influence cumulative gold recovery and grade in flotation?

How do non-linear effects and feature interactions impact process performance?

How can SHAP-based interpretability provide actionable guidance for process optimization in small-data scenarios?

Based on these research questions, we propose the following hypotheses:

**H1.**  *Power, head grade, and time are the dominant features influencing cumulative recovery and grade.*

**H2.**  *Incorporating interaction terms through polynomial features enhances interpretability and reveals additional explanatory relationships beyond individual parameters.*

## 2. Methodology

### 2.1. Data Collection and Preprocessing

**Scheme 1:**
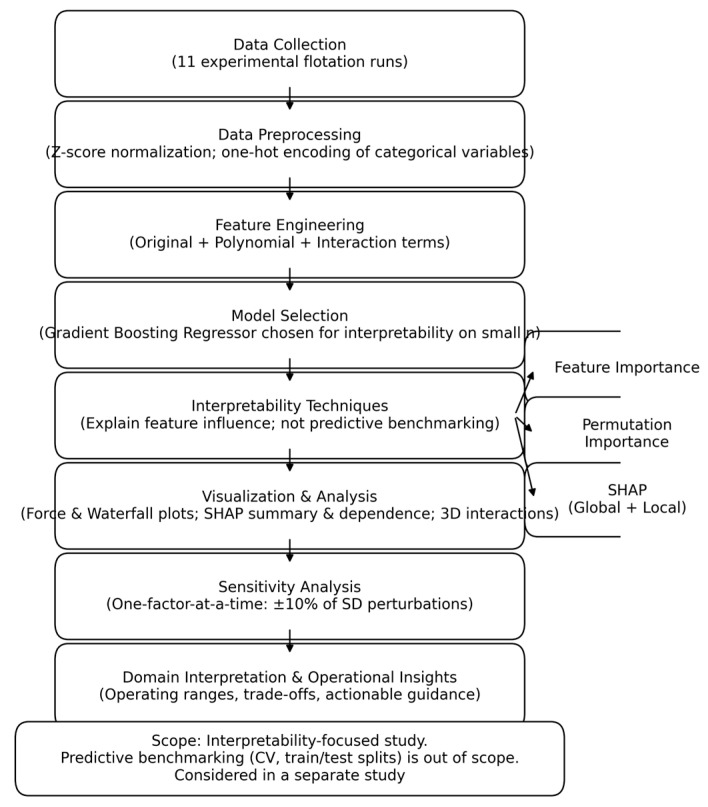
Methodology design: the machine learning analyses were implemented in **Python** (scikit-learn 1.3). We used **scikit-learn**: for model development, including Gradient Boosting Regressor and Random Forest, as well as preprocessing (e.g., z-score normalization, one-hot encoding). We used **SHAP (SHapley Additive exPlanations)** for global and local interpretability of model predictions, including summary plots, dependence plots, and force/waterfall visualizations. We used **matplotlib and seaborn** for data visualization, including pair plots, SHAP plots, and sensitivity analysis. We used **NumPy and pandas** for data manipulation and statistical analysis. These tools were selected for their robustness, compatibility with ensemble models, and widespread use in explainable machine learning workflows.

The dataset used in this study was obtained from controlled laboratory experiments involving gold ore from the Ballarat gold mine (Table 1) [10]. The experiments represent industrially relevant operating conditions. Although the dataset comprises only 11 experimental runs, this reflects the practical constraints of conducting controlled flotation tests with Ballarat ore under laboratory conditions. Such experiments are time- and resource-intensive, and small datasets are common in mineral processing research. The use of explainable machine learning techniques—such as SHAP and permutation importance—enables meaningful insights even in low-data scenarios by focusing on interpretability rather than predictive generalization. The consistency of results across models and alignment with domain knowledge further support the validity of conclusions drawn from this dataset. The gold ore was processed using a jaw crusher, followed by VSI and HPGR crushers operating at different speeds, producing particle sizes ranging from +300 µm to −600 µm [11]. The independent variables included head grade, particle size, crushing time, and Power consumption, while the dependent variables were cumulative gold recovery and grade (Table 2). Descriptive flowchart of methodology design is illustrated in Scheme 1.

Categorical variables, such as the type of collector used (Potassium Amyl Xanthate, PAX, and Di Butyl Dithiophosphate, DSP), and the crushers used (High Pressure Grinding Rolls High Speed (HPGR HS), High Pressure Grinding Rolls Low Speed (HPGR LS), Vertical Shaft Impactor (VSI)) were encoded using one-hot encoding. One-hot encoding was chosen to prevent any ordinal relationships from being introduced, as the collectors (PAX and DSP) or crushers have no inherent order. Although target encoding was considered, it was ultimately avoided to prevent data leakage. Future work may explore target encoding to capture potential relationships between collector type and cumulative gold recovery.

To ensure consistency across different crushing methods (VSI, HS-HPGR, LS-HPGR), the dataset was standardized using z-score normalization across features. The z-score is computed as

$$Z=\frac{X-\mu }{\sigma }$$

where

- (*X*) is the original data point;
- (μ) is the mean of the feature;
- (σ) is the standard deviation of the feature.

When data is z-score normalized, all features are transformed to have the same scale (mean 0, standard deviation 1), making it easier to compare and analyze different variables.

### 2.2. Descriptive Statistics

Table 3 summarizes the descriptive statistics of the dataset, highlighting the variability in key parameters such as size, Power, time, head grade, cumulative recovery, and cumulative grade. The data spans a wide range, with cumulative recovery and grade showing significant variability (standard deviations of 4.66% and 37.09%, respectively), indicative of diverse experimental conditions and outcomes. These statistics provide valuable insights into the data distribution, central tendencies, and variability, forming a robust foundation for subsequent analyses.

### 2.3. Visualizing the Data

**Figure 1:**
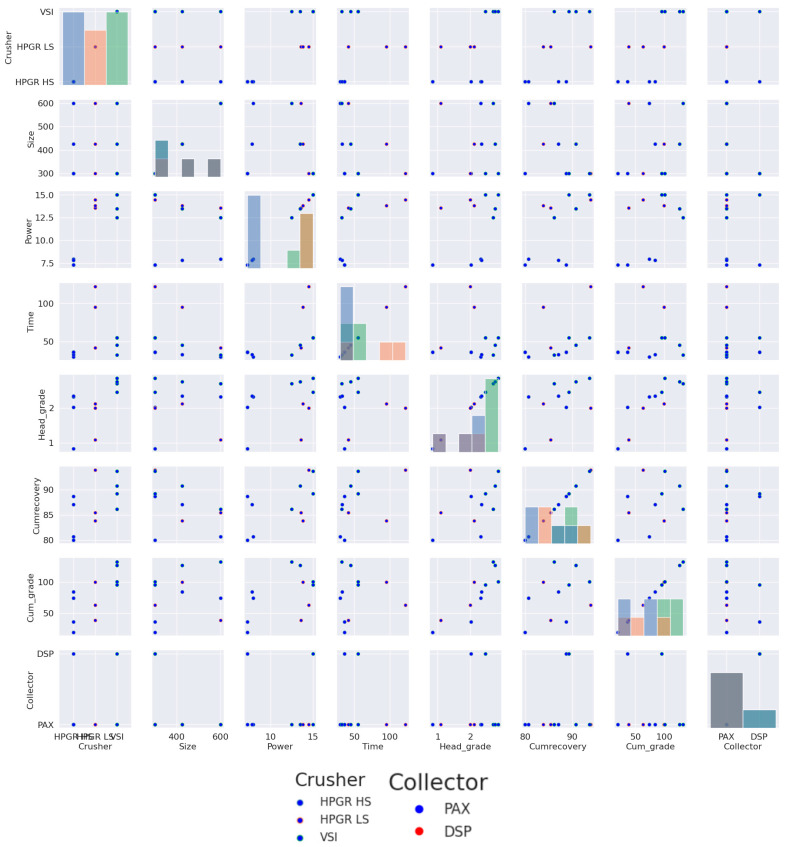
Pair plot visualization of the data in Table 2.

The pair plot (Figure 1) visualizes relationships among crusher type, particle size, power, processing time, head grade, cumulative recovery, cumulative grade, and collector type. Diagonal plots show variable distributions, while off-diagonal scatter plots illustrate pairwise relationships. Color differentiates crusher types, with collector types shown as blue (PAX) and red (DSP). Key trends include positive correlations between processing time and recovery, head grade and cumulative grade, and particle size and power. Annotations highlight these relationships for clarity.

#### 2.3.1. Specific Relationships Between Variables

Processing Time (time) and Cumulative Recovery: There is likely a positive correlation between processing time and cumulative recovery, particularly evident for the HPGR LS crusher (indicated by the upward trend of red dots in the corresponding scatter plot). This suggests that longer processing times generally lead to higher cumulative recovery, which aligns with the domain knowledge that extended processing allows for greater extraction of valuable minerals.

Head Grade and Cumulative Grade: A positive correlation is expected between head grade and cumulative grade, potentially more pronounced for the VSI crusher (indicated by a steeper upward trend of green dots). This is consistent with the understanding that higher head grades in the ore generally result in higher cumulative grades in the final product.

Particle Size (size) and Power Consumption (Power): A potential positive correlation exists between particle size and Power consumption, especially for the HPGR crushers (indicated by an upward trend of red and blue dots). This suggests that crushing larger particles requires more Power, aligning with the domain knowledge that larger particles demand more energy for size reduction.

Head Grade and Cumulative Recovery: While the primary influence of head grade is on cumulative grade, there might be a subtle positive correlation between head grade and cumulative recovery, particularly for the HPGR HS crusher (indicated by a slight upward trend of blue dots). This could indicate that higher head grades might indirectly improve recovery by facilitating faster liberation of valuable minerals.

Crusher Type and Cumulative Recovery/Cumulative Grade: The color coding reveals potential differences in cumulative recovery and grade across crusher types. For example, the HPGR LS crusher (red dots) exhibits higher cumulative recovery at longer processing times compared to other crushers. Similarly, the VSI crusher (green dots) achieve higher cumulative grades for certain head grade levels.

Collector Type and Cumulative Recovery/Cumulative Grade: The blue and red points representing ‘PAX’ and ‘DSP’ collectors, respectively, show variations in their relationships with cumulative recovery and grade. Analyzing these patterns can reveal if different collector types influence recovery or grade differently.

Non-linear Relationships: There might be an optimal processing time beyond which further increases have diminished returns on cumulative recovery. Similarly, an optimal head grade range might exist for maximizing cumulative grade.

#### 2.3.2. Limitations

Correlation vs. Causation: The observed relationships indicate correlations, not necessarily causal relationships. Further investigation is needed to establish causality.

Limited Data: The interpretations are based on the observed data, which might have limitations in terms of sample size and representation. The domain-specific interpretations can be validated by experts in the field.

Overall, the plot helps in identifying correlations, distributions, and potential influences of ‘Crusher’ and ‘Collector’ types on the variables being analyzed [12]. The pair plot provides valuable insights for understanding the interdependencies between crusher operating parameters and their relationship to ore characteristics and collector choice. By visualizing these relationships, domain experts can confirm existing knowledge, identify unexpected patterns, and generate new hypotheses about how these factors interact. For instance, observing the impact of crusher type on Power consumption for different ore sizes could guide optimization efforts for energy efficiency. Similarly, identifying patterns related to collector type might lead to improved strategies for material recovery and product quality. The plot thus facilitates data-driven decision-making and process refinement within the domain.

### 2.4. Features Transformation

Description of Features Used in the Analysis is presented in Table 4. We considered the transformation of features to enhance model interpretability and to explore domain-relevant interactions. Original features (e.g., ‘size’, ‘Power’, ‘time’, ‘head_grade’, ‘Crusher_HPGR HS’, ‘Crusher_HPGR LS’, ‘Crusher_VSI’, ‘Collector_DSP’, ‘Collector_PAX’) represent the raw, untransformed inputs. Ensemble methods like Random Forests and Gradient Boosting can capture non-linear relationships and interactions effectively, leveraging their structure without requiring explicit feature transformations.

However, polynomial features extend the feature space by introducing higher-order and interaction terms (e.g., x^2^, x_1_·x_2_). These are particularly beneficial for models that assume linearity or have limited capacity to learn interactions directly, such as linear regression or neural networks. In this study, polynomial features were included not for model optimization, but to explicitly represent hypothesized interactions grounded in mineral processing domain knowledge—such as the combined effect of head grade and collector type, or Power and processing time.

This approach enables interpretable machine learning techniques like SHAP and permutation importance to uncover nuanced relationships that may not be evident from original features alone. It supports the study’s goal of understanding why certain variables influence cumulative recovery and grade, rather than simply predicting outcomes.

While ensemble methods can implicitly model interactions, the inclusion of polynomial features allows for direct control and visibility over these effects, which is crucial for validating domain-specific hypotheses [13]. The full list of polynomial features used in this study is provided below to ensure transparency and reproducibility.

Original Features: [‘size’, ‘Power’, ‘time’, ‘head_grade’, ‘Crusher_HPGR HS’, ‘Crusher_HPGR LS’, ‘Crusher_VSI’, ‘Collector_DSP’, ‘Collector_PAX’].

Polynomial Features: [‘size’, ‘Power’, ‘time’, ‘head_grade’, ‘Crusher_HPGR HS’, ‘Crusher_HPGR LS’, ‘Crusher_VSI’, ‘Collector_DSP’, ‘Collector_PAX’, ‘size^2^’, ‘size Power’, ‘size time’, ‘size head_grade’, ‘size Crusher_HPGR HS’, ‘size Crusher_HPGR LS’, ‘size Crusher_VSI’, ‘size Collector_DSP’, ‘size Collector_PAX’, ‘Power^2^’, ‘Power time’, ‘Power head_grade’, ‘Power Crusher_HPGR HS’, ‘Power Crusher_HPGR LS’, ‘Power Crusher_VSI’, ‘Power Collector_DSP’, ‘Power Collector_PAX’, ‘time^2^’, ‘time head_grade’, ‘time Crusher_HPGR HS’, ‘time Crusher_HPGR LS’, ‘time Crusher_VSI’, ‘time Collector_DSP’, ‘time Collector_PAX’, ‘head_grade^2^’, ‘head_grade Crusher_HPGR HS’, ‘head_grade Crusher_HPGR LS’, ‘head_grade Crusher_VSI’, ‘head_grade Collector_DSP’, ‘head_grade Collector_PAX’, ‘Crusher_HPGR HS^2^’, ‘Crusher_HPGR HS Crusher_HPGR LS’, ‘Crusher_HPGR HS Crusher_VSI’, ‘Crusher_HPGR HS Collector_DSP’, ‘Crusher_HPGR HS Collector_PAX’, ‘Crusher_HPGR LS^2^’, ‘Crusher_HPGR LS Crusher_VSI’, ‘Crusher_HPGR LS Collector_DSP’, ‘Crusher_HPGR LS Collector_PAX’, ‘Crusher_VSI^2^’, ‘Crusher_VSI Collector_DSP’, ‘Crusher_VSI Collector_PAX’, ‘Collector_DSP^2^’, ‘Collector_DSP Collector_PAX’, ‘Collector_PAX^2^].

Note on Feature Selection for Future Work:

Although feature selection was not within the scope of this study, future work may explore techniques such as Lasso regression or Permutation-based Conditional Importance (PCI) to refine the feature set, especially when larger datasets are available. These methods could help balance model complexity with interpretability by identifying the most impactful interactions.

### 2.5. Modeling Approach

Ensemble methods like Random Forests and Gradient Boosting can capture non-linear relationships and interactions effectively, leveraging their structure without requiring explicit feature transformations [14]. A Gradient Boosting Regressor is robust in handling complex non-linear relationships and high-dimensional data which are common in this field of mineral processing. The model builds sequential decision trees, with each tree correcting the errors of the previous one, allowing it to model complex interactions between variables such as particle size and head grade. This sequential approach makes Gradient Boost Regression particularly effective in capturing the intricacies of flotation recovery processes, in identifying the features having most impact on the targets (Cumulative Recovery and Cumulative Grade).

Integration of feature importance, permutation analysis, and SHAP analysis, allows us to gain a comprehensive understanding of the factors influencing flotation recovery and grade. This holistic approach allows us to identify key features and interactions, optimize the flotation process, and improve the predictive Power and interpretability of the model.

The Gradient Boosting Regressor was chosen for its robustness with small datasets and compatibility with SHAP interpretability techniques. For feature importance, Random Forest method is included for comparison. While performance evaluation through metrics like R^2^ or RMSE and cross-validation is essential for generalization, this work prioritizes interpretability. Predictive validation will be reported in a subsequent study focusing on model predictability and optimization.

### 2.6. Feature Importance

This method measures the contribution of each feature to the model’s predictions by evaluating the decrease in a performance metric—such as mean square error or R^2^—when the feature is used to split the data. It offers a quick and intuitive understanding of which features are most influential in the model by highlighting their relative importance based on how much they help reduce prediction error. However, it may not effectively capture complex interactions between features and can be biased toward features with more levels or higher cardinality.

### 2.7. Permutation Analysis

Permutation analysis validates feature importance by randomly shuffling the values of each feature and measuring the resulting impact on model performance. This approach helps confirm the significance of features such as Power, head grade, and collectors in predicting recovery and grade. It evaluates the importance of a feature by observing the decrease in a performance metric—like accuracy or R^2^—when the feature’s relationship with the target variable is disrupted. This method provides a more robust measure of feature importance by directly assessing each feature’s effect on the model’s performance and accounting for interactions between features. It also includes standard deviation to gauge the stability of importance scores. However, permutation analysis is computationally intensive, requiring multiple evaluations of the model, and can be sensitive to the choice of performance metric and the number of permutations.

### 2.8. SHAP Analysis

SHAP (SHapley Additive exPlanations) values provide a detailed explanation of the impact of each feature on the model’s predictions. SHAP analysis can be used to visualize the contribution of Power, head grade, and collectors to the predicted recovery and grade, highlighting the importance of these features and their interactions. Understanding the contributions of individual variables to model predictions is crucial for optimizing recovery and grade in flotation studies. SHAP insights can be used to adjust operational parameters in real-time, ensuring optimal conditions for recovery and grade. It is possible to Identify key features that impact recovery and grade and use this information to schedule maintenance activities that prevent process disruptions. Also used for quality Assurance to monitor feature values and their impacts to maintain consistent product quality. SHAP values provide a robust framework for interpreting these contributions by offering both global and local explanations.

### 2.9. Analysis of Feature and Permutation Importance for Cumulative Recovery and Grade

**Figure 2:**
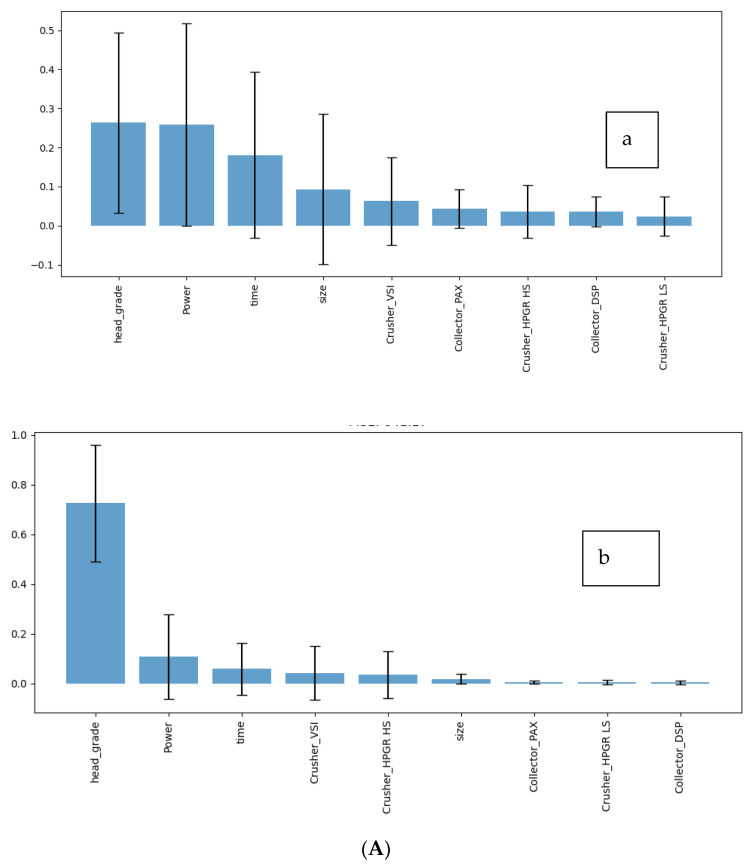

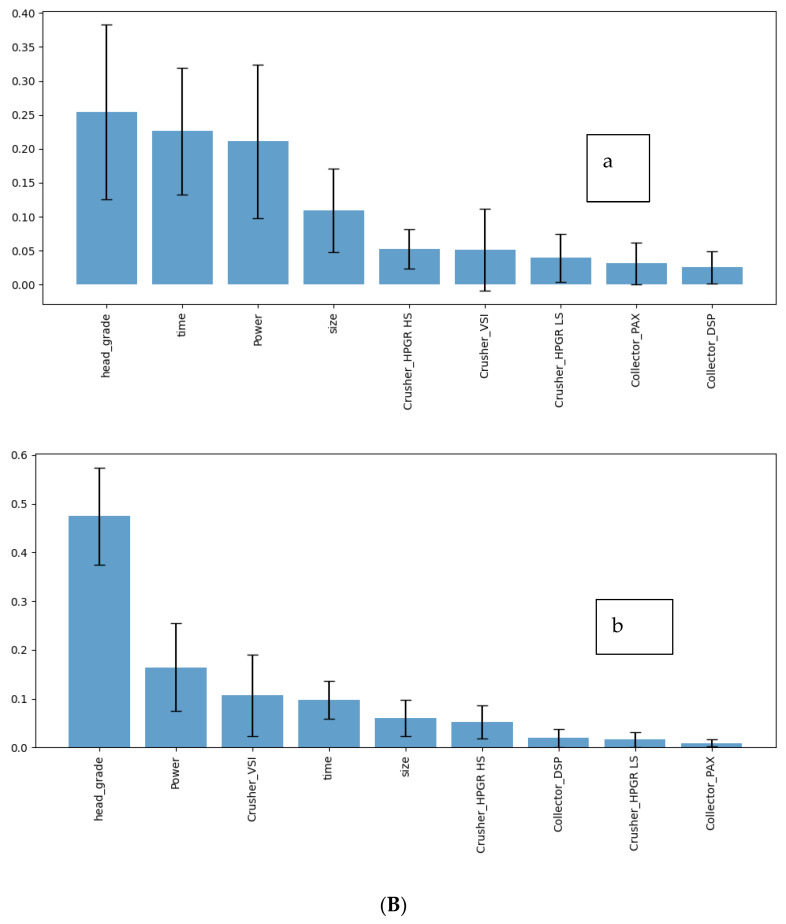
(**A**) Feature importance using gradient boosting for original features: (**a**) cumulative recovery, 32.74; (**b**) cumulative grade, 841.17. (**B**) Feature importance using random forest for original features: (**a**) cumulative recovery, MSE 23.72; (**b**) cumulative grade, MSE 668.19.

**Figure 3:**
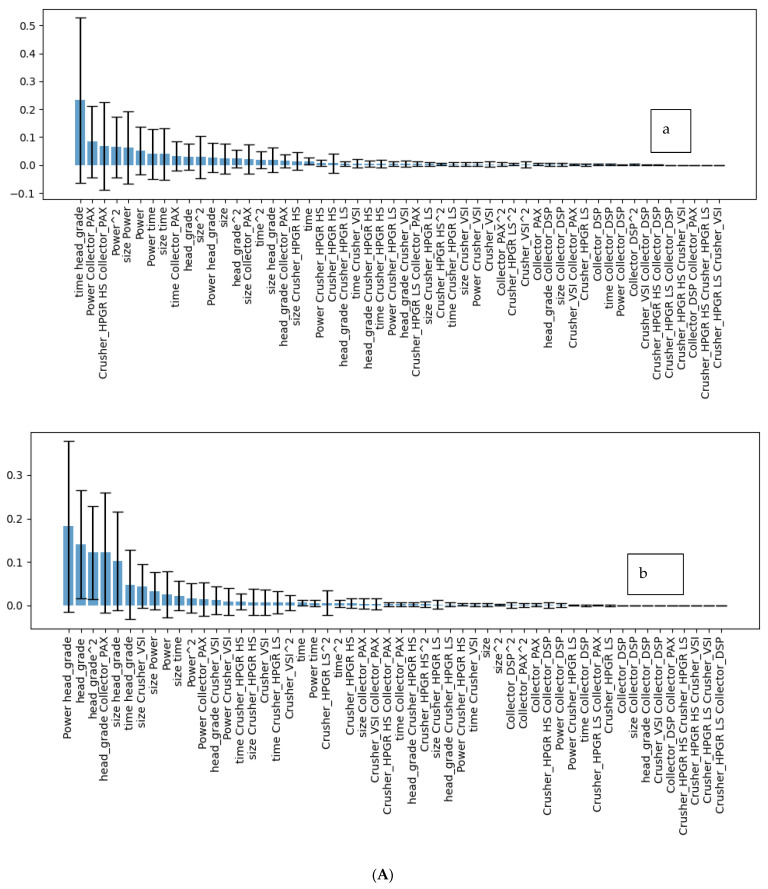

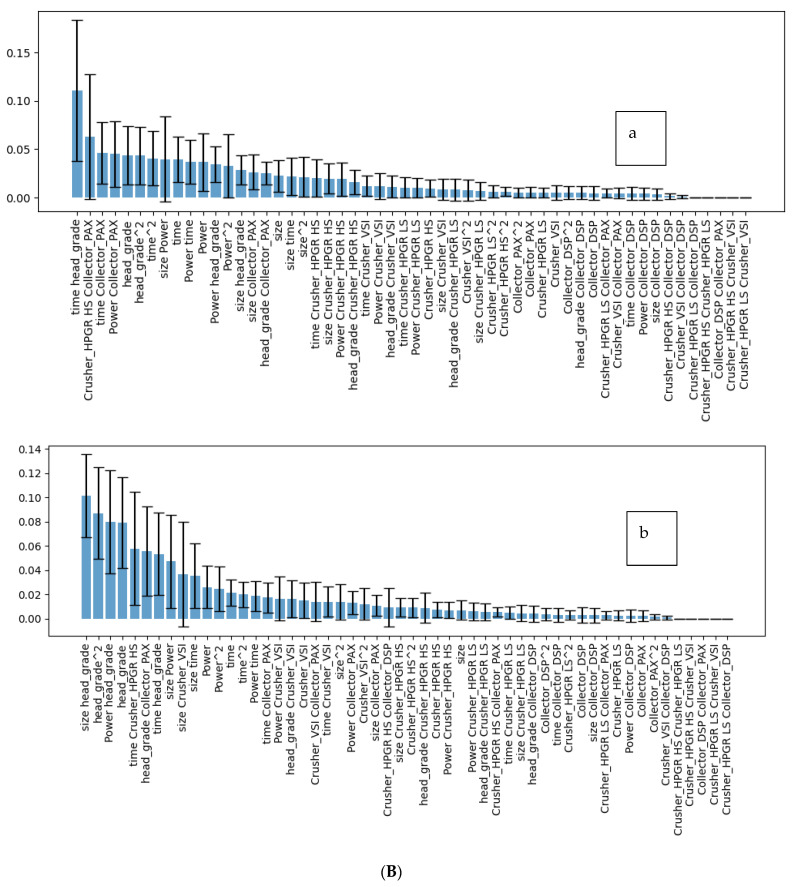
(**A**) Feature importance using gradient for polynomial features: (**a**) cumulative recovery, MSE 30.35; (**b**) cumulative grade, MSE 633.67. (**B**) Feature importance using random forest for polynomial features: (**a**) cumulative recovery, MSE 23.30; (**b**) cumulative grade, MSE 824.62.

**Figure 4:**
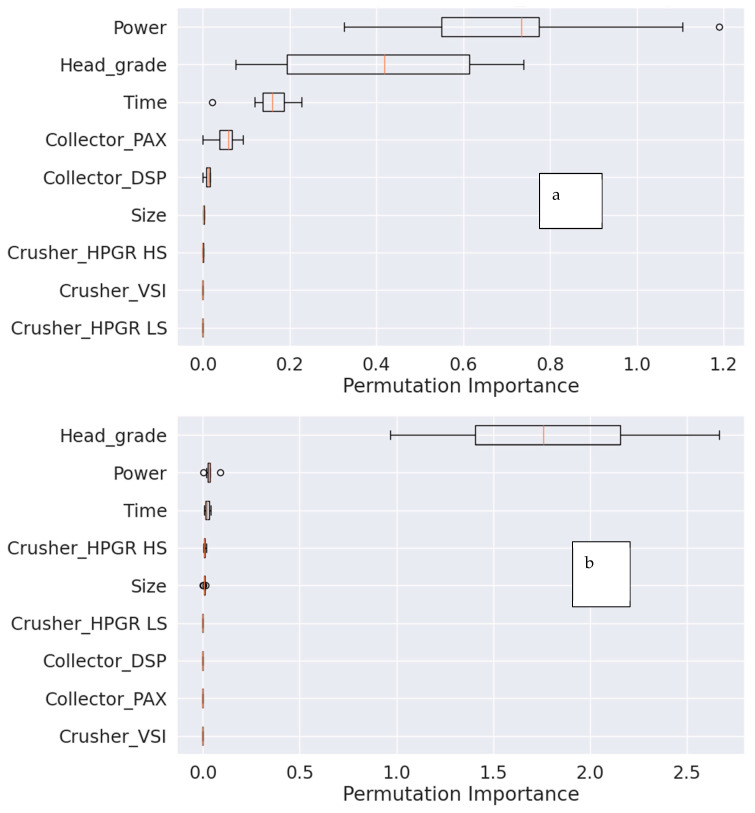
Permutation importance for original features: (**a**) cumulative recovery, (**b**) cumulative grade.

**Figure 5:**
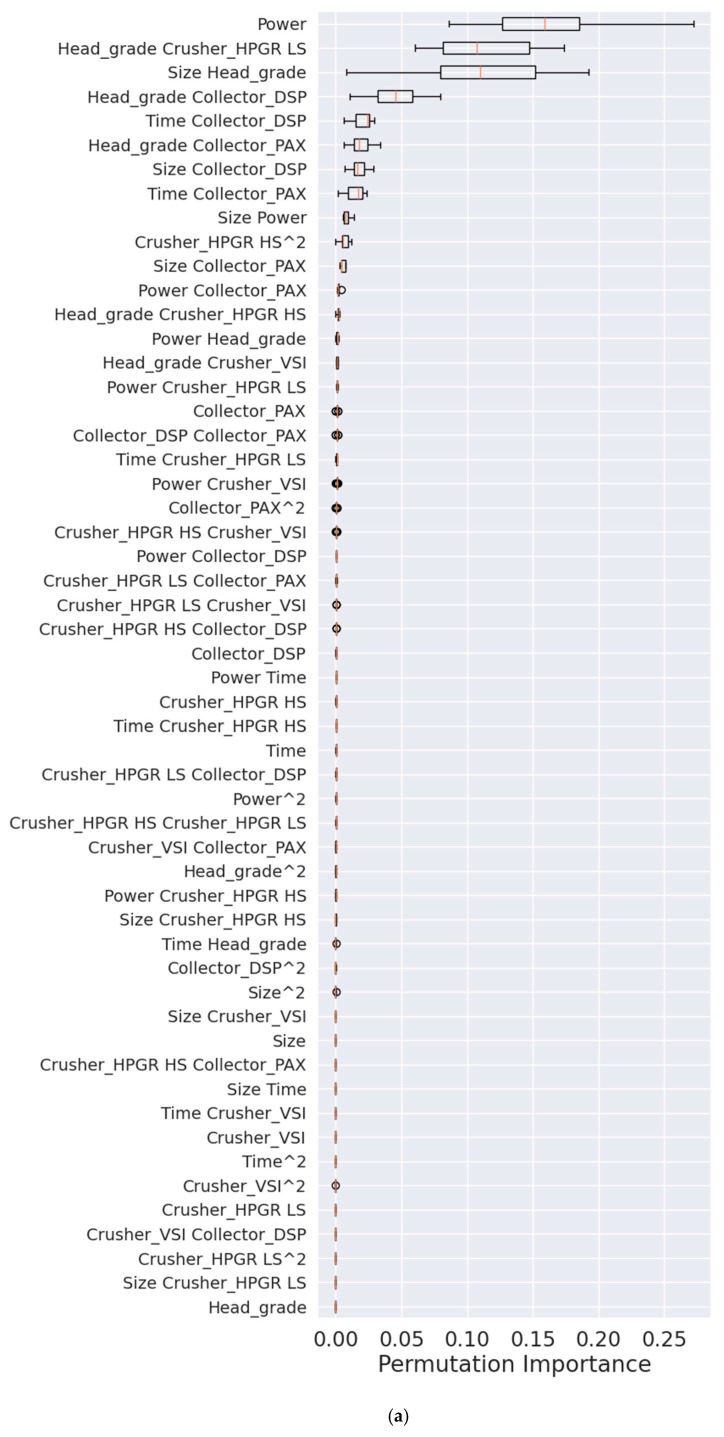

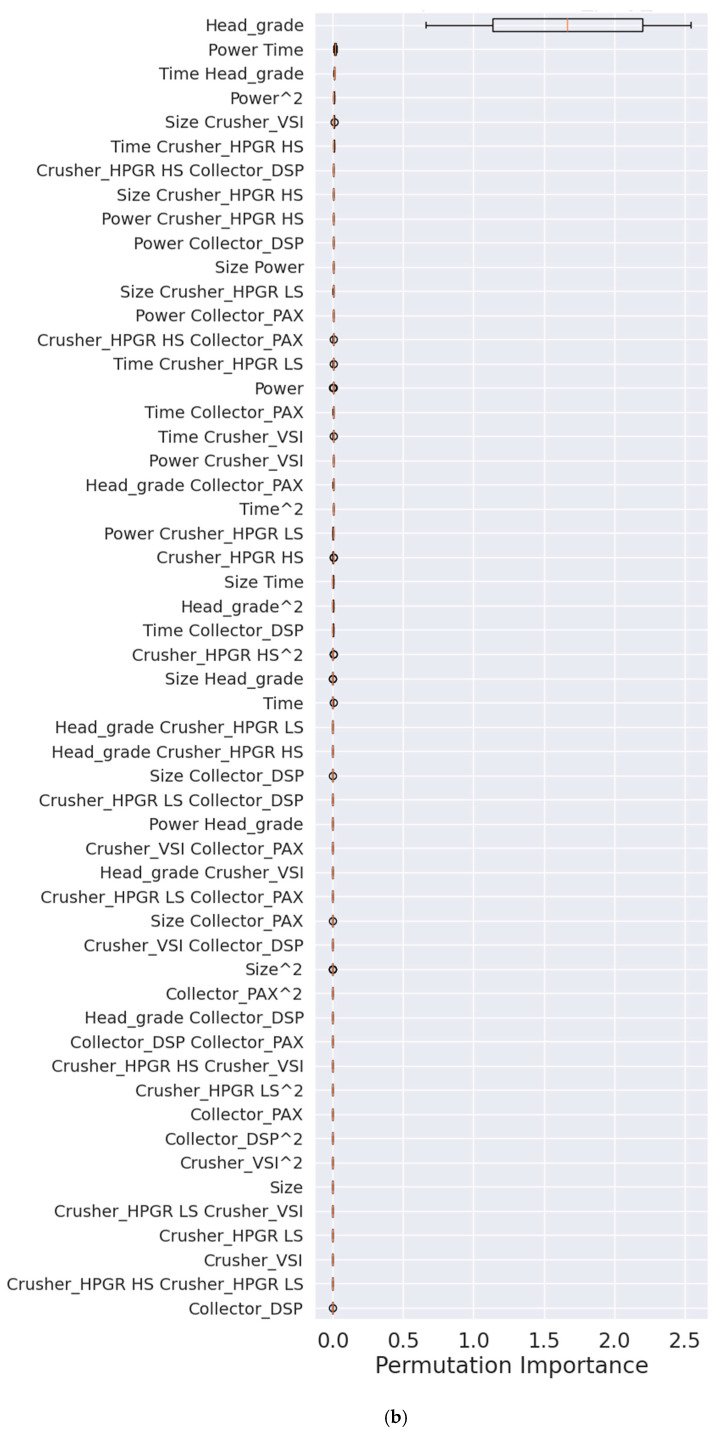
Permutation importance for polynomial features: (**a**) cumulative recovery (**b**) cumulative grade.

Cumulative Recovery (y_1_) Figure 2, Figure 3, Figure 4 and Figure 5 illustrate the feature and permutation importance plots for cumulative recovery for original and polynomial features, respectively.

Original Features:

Among the original features, Power stands out as the most significant, with a feature importance of 0.476 and a permutation importance of 0.72 (standard deviation: 0.252), highlighting its crucial role in determining cumulative recovery. Head Grade also plays a key role, with a feature importance of 0.248 and permutation importance of 0.42 (Std: 0.24), reflecting the quality of the ore being processed. Time shows moderate influence, with importance scores of 0.123 (feature) and 0.155 (permutation, Std: 0.054), suggesting that the duration of the crushing process affects recovery, though less than Power and head grade. Collector PAX has a notable impact, with scores of 0.111 (feature) and 0.054 (permutation, Std: 0.029), while Collector DSP shows a smaller but still relevant effect, with scores of 0.036 (feature) and 0.011 (permutation, Std: 0.006). Size has minimal influence, with importance values of 0.006 (feature) and 0.002 (permutation, Std: 0.0006). Lastly, Crusher Types (HPGR HS, HPGR LS, VSI) exhibit very low importance values, indicating a negligible direct impact on recovery.

Polynomial Features:

In the analysis of interaction features, Power remains a significant factor with a feature importance of 0.296 and a permutation importance of 0.167 (standard deviation: 0.056), although its influence is reduced compared to when considered as an original feature. The interaction between Size and Head Grade shows equal importance scores of 0.111 for both feature and permutation (Std: 0.054), highlighting a meaningful relationship. The Time and Collector-DSP interaction has a feature importance of 0.062 and permutation importance of 0.021 (Std: 0.008), indicating a notable effect. Similarly, the interaction between Head Grade and Crusher HPGR LS demonstrates strong influence with scores of 0.12 (feature) and 0.112 (permutation, Std: 0.039). The combined effect of Head Grade and Collector DSP is also significant, with importance values of 0.11 (feature) and 0.044 (permutation, Std: 0.0198). Other interactions, such as Power with Crusher VSI and Time with Crusher HPGR HS, show smaller but still relevant importance values, suggesting complex relationships among features.

Cumulative Grade (y_2_) Figure 2, Figure 3, Figure 4 and Figure 5 illustrate the feature and permutation importance plots for cumulative grade for original and polynomial features, respectively.

Original Features:

- Head Grade: Dominates with a feature importance of 0.888 and permutation importance of 1.806 (Std: 0.512), underscoring its critical role in determining cumulative grade [15].
- Time: Importance of 0.044 (feature) and 0.022 (permutation, Std: 0.0120), indicating a moderate impact.
- Power: Importance of 0.040 (feature) and 0.033 (permutation, Std: 0.021), showing a similar level of influence as time.
- Size: Importance of 0.0146 (feature) and 0.007 (permutation, Std: 0.003), suggesting a minor effect.
- Crusher HPGR HS: Importance of 0.012 (feature) and 0.008 (permutation, Std: 0.0038), indicating some relevance.
- Other Features (Crusher HPGR LS, VSI, Collectors): Very low importance values, suggesting minimal direct impact on grade.

Polynomial Features:

- Head Grade: Still highly significant with a feature importance of 0.73 and permutation importance of 1.646 (Std: 0.646), though slightly reduced compared to the original feature.
- Power Time: Importance of 0.045 (feature) and 0.015 (permutation, Std: 0.007), highlighting the interaction between Power and time.
- Size Crusher VSI: Importance of 0.030 (feature) and 0.0056 (permutation, Std: 0.0023), indicating a notable interaction effect [2,16].
- Time Head Grade: Importance of 0.032 (feature) and 0.008 (permutation, Std: 0.002), showing the combined influence of time and head grade.
- Other Interactions: Various other interactions (e.g., Power Crusher VSI, Time Crusher HPGR HS) show smaller but notable importance values, indicating complex relationships between features.

The analysis reveals that while certain features like Power and head grade are consistently important across both original and polynomial contexts, the inclusion of polynomial features uncovers significant interaction effects that are not apparent in the original feature set. For cumulative recovery, interactions such as size head grade and head grade collector DSP emerge as critical factors. Similarly, for cumulative grade, interactions like Power time and size crusher VSI become relevant. These findings underscore the value of incorporating polynomial features in machine learning analyses, particularly for small datasets where capturing complex relationships can significantly enhance model interpretability and predictive Power.

Permutation importance largely reinforces the findings from the feature importance analysis. Both methods highlight the critical role of features like Power, head grade, and collectors (PAX and DSP) in determining cumulative recovery and grade.

However, permutation importance adds value by providing:

Stability Assessment: The standard deviation values from permutation importance help assess the stability of the importance scores, indicating how consistently a feature impacts the model’s performance (while permutation importance inherently includes a measure of stability (standard deviation) as part of its calculation, feature importance can also be assessed for stability using additional techniques like bootstrapping or cross-validation).

Robustness: Permutation importance offers a more robust measure by directly evaluating the impact of each feature on the model’s performance, validating the feature importance results.

Interaction Effects: While both methods can capture interactions, permutation importance can sometimes highlight the combined effects of features more clearly by considering the change in performance when a feature is permuted.

Comparative results across Gradient Boosting and Random Forest (Figure 2B and Figure 3B) models are summarized in Table 5. Random Forest consistently outperformed Gradient Boosting in predicting cumrecovery, especially with polynomial features. For Cumulative grade, Gradient Boosting with polynomial features gave the best performance, suggesting it may better capture complex interactions. Polynomial features improved performance in most cases but also increased model complexity. Error bars show that Random Forest had more stable feature importance across runs, especially for cumrecovery.

Gradient Boosting consistently outperformed Random Forest and ExtraTrees in terms of validation R^2^ and RMSE, while SVR exhibited unstable performance due to sensitivity to kernel hyperparameters and the small dataset size. These findings support the use of Gradient Boosting as the primary model in this study, while demonstrating that tree-based ensemble methods generally provide more robust performance than kernel-based methods in small, heterogeneous mineral processing datasets.

#### Domain Considerations

Effect of Power Consumption on Flotation Recovery

The Power and type of crushers used in mineral processing can significantly influence the efficiency of subsequent flotation processes, affecting both recovery rates and concentrate grades. High Voltage Electrical Pulse (HVEP) crushing has been shown to produce coarser particles with fewer slimes compared to traditional mechanical crushing methods. This selective fragmentation enhances mineral liberation, leading to improved flotation performance. For instance, in the flotation of sulfide minerals crushed by an electric pulse crusher, recovery rates were observed to be 10.6% higher, and the sulfur grade in the flotation residue was approximately 58% lower than in samples subjected to mechanical crushing. Additionally, the flotation kinetics constant increased by 37.5% with HVEP crushing, indicating a more efficient flotation process [17].

Particle shape and surface roughness, which are influenced by the crushing method, also play a crucial role in flotation efficiency. Variations in these physical properties can affect particle-bubble interactions, thereby impacting both grade and recovery in flotation processes. The usage of impact crushers produces more liberated particles during size reduction [18]. The shape of particles, which depends on the grinding time, reportedly improved the recovery of chromite in a binary mixture of serpentinite as the roundness decreased, becoming angular. Similarly, the recovery improved as the shape attained higher angularity [19].

The energy input during crushing can affect the particle size distribution, which in turn influences flotation recovery. Excessive energy input may lead to over-grinding, producing ultra-fine particles that can adversely affect flotation performance. Therefore, optimizing the crushing process is essential to achieve a balance between adequate mineral liberation and the prevention of excessive fines that could hinder flotation efficiency. It was reported that recoveries could be increased and grade of concentrates improved by removing freed mineral ahead of final fine grinding [20].

Particle Characteristics Produced by HPGR at High and Low Speeds, and by VSI

The choice between high-speed HPGR, low-speed HPGR, and VSI depends on the balance required between liberation, surface characteristics, and flotation performance. Fine-tuning operating parameters is essential to optimize outcomes for specific ore types.

High-Speed HPGR:

Particle Liberation: Produces finer particles with a higher degree of mineral liberation. Enhanced exposure of grain boundaries due to higher shearing forces.

Particle Shape: Generates relatively angular particles with rough surfaces. Favors increased surface area, enhancing collector adsorption in flotation.

Crack Formation: Induces more micro-cracks, which improve flotation recovery.

Energy Efficiency: Higher speed leads to more efficient energy use but may result in over-grinding, especially for softer minerals.

Low-Speed HPGR:

Particle Liberation: Produces coarser particles compared to high-speed operation. Lower shearing leads to less mineral liberation.

Particle Shape: Particles are blockier with fewer fines. Suitable for size-sensitive processes but less effective in flotation where fine particles are advantageous.

Crack Formation: Generates fewer micro-cracks, potentially reducing flotation efficiency.

Energy Efficiency: More energy consumption per ton of material due to lower throughput.

VSI Characteristics:

Particle Liberation: Effective at producing finely liberated particles through impact and attrition. Ensures uniform particle size distribution.

Particle Shape: Generates highly angular particles with smoother surfaces compared to HPGR. The smoother surface can reduce collector attachment efficiency.

Particle Size Distribution: Produces a wide range of particle sizes. May lead to challenges in flotation if ultra-fines are prevalent.

Wear Characteristics: Higher wear rates on VSI components due to the impact forces.

Comparative Impact on Flotation:

High-Speed HPGR: High liberation and surface roughness enhance flotation recovery. May lead to challenges with ultra-fines affecting grade.

Low-Speed HPGR: Generates coarser particles, which may reduce flotation recovery but improve concentrate grade.

VSI: Provides angular particles favorable for bubble-particle attachment but can result in excessive fines, reducing overall recovery. However, in the present study, though involved several passes to produce the required size fraction, only the coarser fraction was crushed, resulting in a greater proportion of coarser particles.

Effect of Collectors PAX and DSP on Gold Recovery and Grade

The effect of collectors such as PAX (Potassium Amyl Xanthate) and DSP (Di thiophosphate) on gold recovery and grade during flotation processes is significant and depends on various factors, including particle size, mineralogy, pulp chemistry, and flotation conditions [21,22].

PAX (Potassium Amyl Xanthate):

Effect on Gold Recovery: PAX is highly effective in recovering gold when it is associated with sulfide ores or when gold is present as a locked particle within sulfide minerals. PAX’s strong collector properties enhance gold flotation, leading to higher recovery rates. It is particularly effective for coarse gold particles as it can form a strong hydrophobic layer that allows them to be effectively separated during flotation.

Effect on Gold Grade: The downside of PAX is that it is less selective. It can also float gangue minerals like pyrite, leading to a decrease in concentrate grade. This is particularly problematic in ores with significant amounts of pyrite or other sulfides that can be floated along with gold. To improve grade, PAX dosage is often carefully controlled, and secondary reagents (such as depressants) are used to minimize the flotation of undesired gangue minerals.

DSP (Dithiophosphate):

Effect on Gold Recovery: DSP is a more selective collector compared to PAX. It tends to have a stronger affinity for base metals and some precious metals like gold, but its effectiveness is more dependent on the mineralogical characteristics of the ore. DSP can recover gold effectively, especially in ores where gold is associated with base metal sulfides. It promotes better selectivity, especially in ores containing a mixture of gold and other metal sulfides.

Effect on Gold Grade: Because DSP is more selective than PAX, it generally results in a higher grade concentrate. This is especially true when the flotation circuit is designed to minimize the flotation of pyrite or other sulfides. As DSP targets sulfide minerals with a stronger affinity, it can reduce the flotation of unwanted gangue, improving the gold concentrate grade. However, the trade-off for higher selectivity is that DSP may not always produce as high a recovery as PAX, particularly when the ore contains high amounts of easily floatable sulfide minerals.

Comparison of PAX and DSP for Gold Recovery and Grade:

PAX: Effective for high recovery, especially in ores with significant amounts of sulfide minerals. Strong collector for coarse gold particles. However, less selective, leading to lower concentrate grade due to the flotation of gangue minerals like pyrite.

DSP: More selective than PAX, improves concentrate grade by avoiding the flotation of gangue minerals, and performs well with gold in sulfide ores. However, lower recovery compared to PAX, particularly in ores with a high proportion of easily floatable sulfides.

## 3. SHAP Analysis for Interpreting Key Variables Influencing Cumulative Recovery and Grade

**Figure 6:**
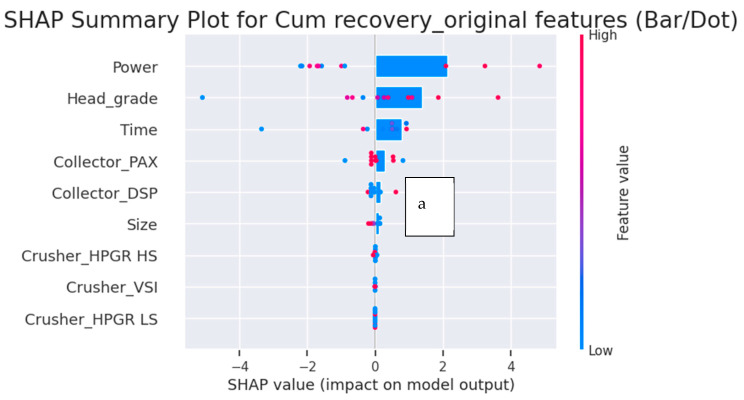

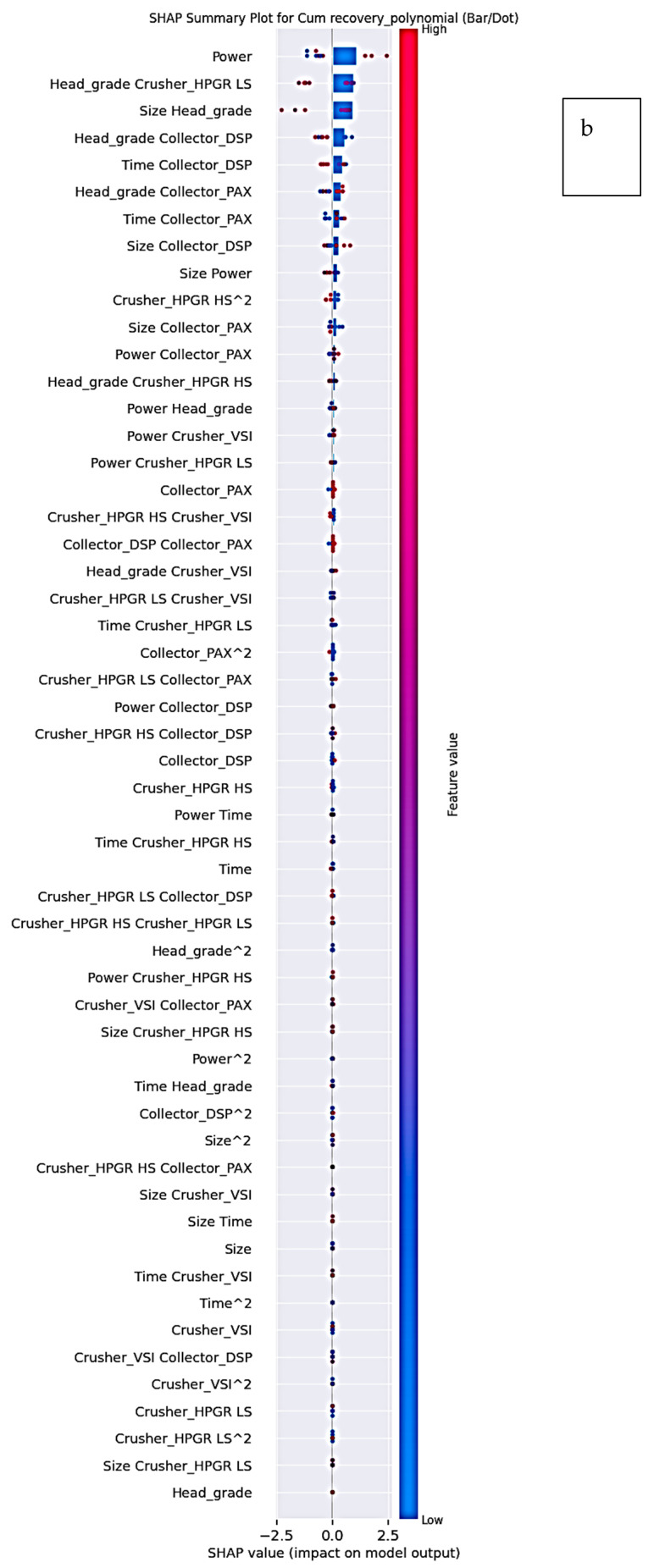
Shap Summary Plot for Cumulative Recovery for (**a**) original features (**b**) polynomial features.

**Figure 7:**
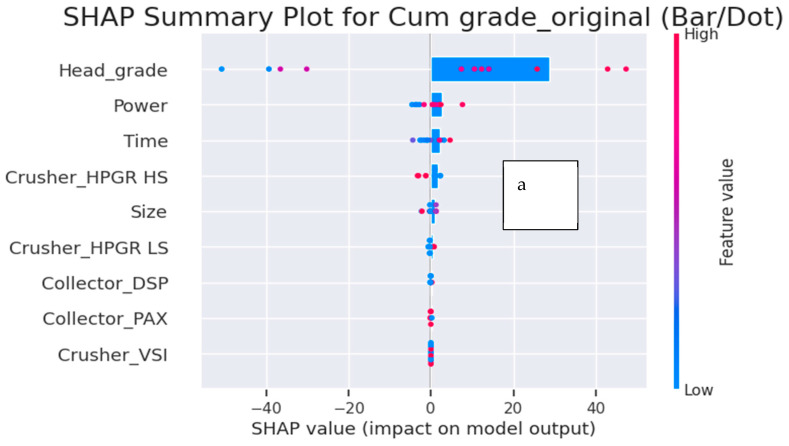

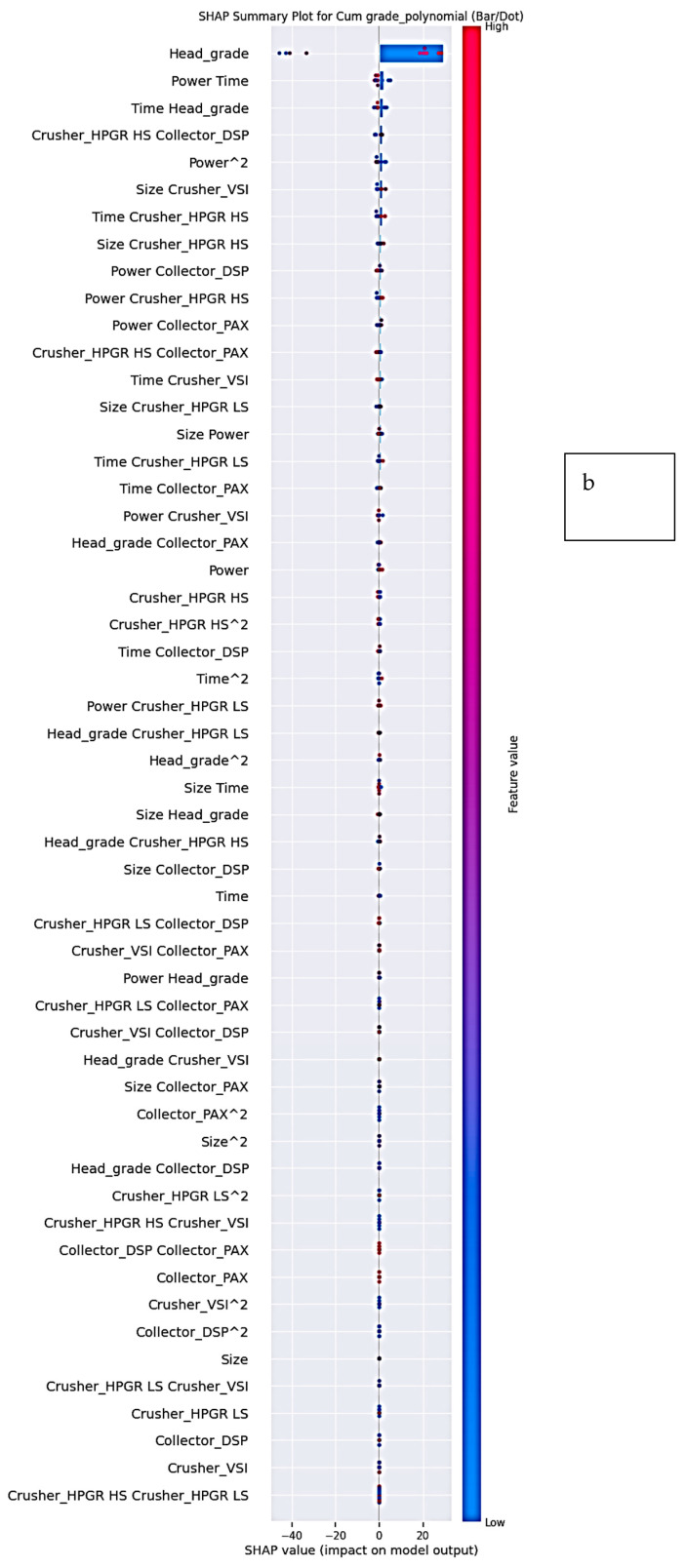
Shap summary plot for cumulative grade for (**a**) original features, (**b**) polynomial features.

Interpretable machine learning methods are crucial for understanding which operational parameters most influence flotation performance. We applied SHAP (SHapley Additive exPlanations) to the Gradient Boosting Regressor to quantify feature contributions for both cumulative recovery (y_1_) and cumulative grade (y_2_). Figure 6a,b and Figure 7a,b present SHAP summary plots (dot and bar) for original and polynomial features.

Key Findings from SHAP Analysis

1. Dominant Features

Power, Head Grade, and Time consistently exhibit the highest SHAP values, confirming their primary role in predicting recovery and grade.

Collector type (PAX vs. DSP) shows moderate influence, with PAX favoring recovery and DSP enhancing grade selectivity.

2. Non-Linear and Interaction Effects

Interaction terms such as Head Grade × Collector and Size × Head Grade appear prominently among polynomial features, indicating that the impact of head grade is conditional on collector choice and particle size distribution.

Higher-order effects (e.g., Power^2^, Time × Head Grade) also contribute to predictive performance, supporting the inclusion of polynomial terms.

3. Directional Influence of Features

Power: Increased Power generally improves recovery but reduces grade, reflecting the trade-off between fine particle liberation and entrainment.

Head Grade: Higher head grades enhance both recovery and grade up to an optimum point, beyond which diminishing returns occur.

Time: Longer grinding improves recovery marginally but decreases grade after a threshold, consistent with over-grinding and entrainment.

Collectors: DSP shows stronger selectivity for grade, while PAX tends to maximize recovery.

4. Robustness and Alignment with Domain Knowledge

SHAP rankings were consistent across original and polynomial feature sets, indicating stable variable importance.

Correlation vs. Causality

It is important to note that SHAP values and permutation importance quantify associations between features and model predictions, not causal relationships. While the observed patterns (e.g., higher Power increasing recovery) align with metallurgical principles, these interpretations should be considered correlative insights rather than definitive causal mechanisms. Establishing causality would require controlled experimental validation or mechanistic modeling. Therefore, the findings presented here serve primarily to guide hypothesis generation and identify operational parameters warranting further study.

## 4. Force Plots and Waterfall Plots: Local Interpretability

Force and waterfall plots enhance local interpretability by illustrating how individual features influence specific predictions of cumulative recovery (y_1_) and cumulative grade (y_2_).

Force Plots show feature impacts using arrows that indicate direction (positive or negative) and magnitude relative to the baseline prediction.

Waterfall Plots provide a sequential breakdown of how each feature moves the prediction from the baseline (E[f(x)]) to the final output (f(x)), making the cumulative contribution of each feature transparent.

These visualizations help identify key drivers for individual instances, complementing global interpretability tools like SHAP summary plots. Table 6 compares the two approaches.

### 4.1. Key Observations from Force and Waterfall Plots

**Figure 8:**
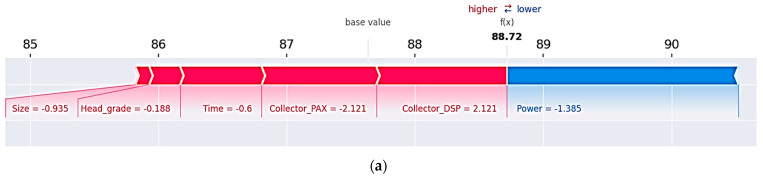

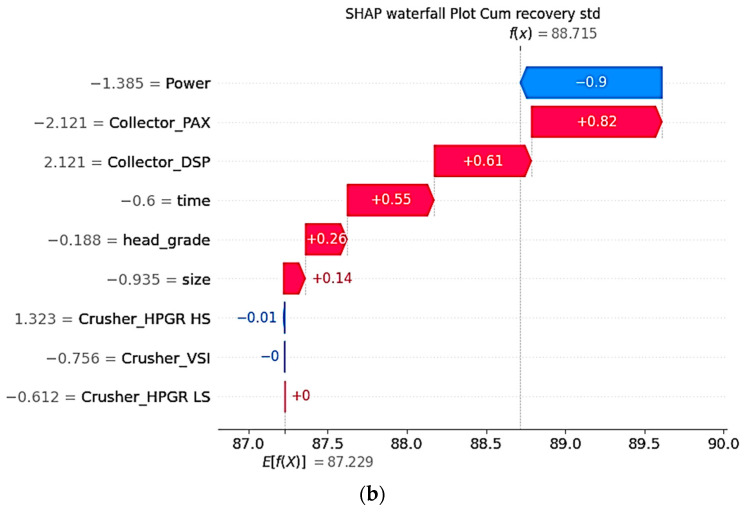
(**a**) Force plot for cumulative recovery, original features. (**b**) Waterfall plot for cumulative recovery original features.

Cumulative Recovery (Original Features)—Figure 8a,b:

Time is the strongest positive contributor (long red bar/arrow), confirming that extended grinding generally improves recovery.

Power shows a slight negative effect (blue), suggesting that beyond a threshold, additional energy input does not enhance recovery and may cause over-grinding.

**Figure 9:**
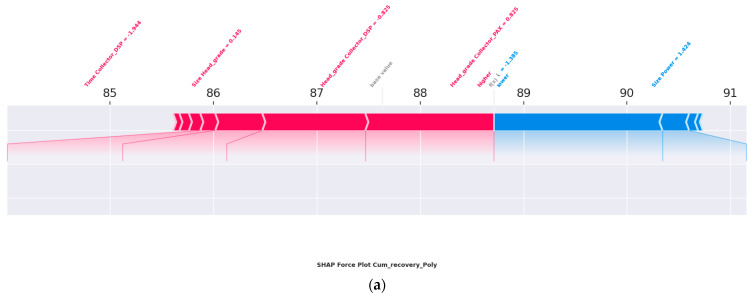

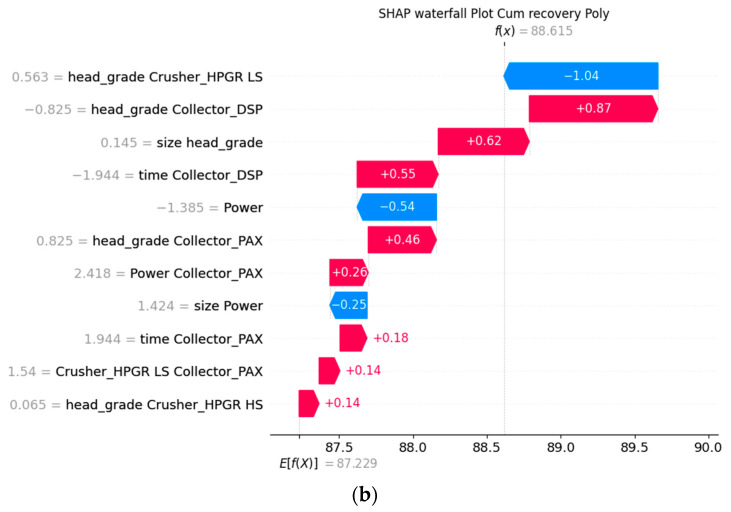
(**a**) Force plot for cumulative recovery polynomial features. (**b**) Waterfall plot for cumulative recovery polynomial features.

Cumulative Recovery (Polynomial Features)—Figure 9a,b:

The term time^2^ has a negative contribution, indicating diminishing returns at excessive grinding durations.

time × head_grade is a significant positive interaction, revealing that high head grades benefit from longer processing times.

**Figure 10:**
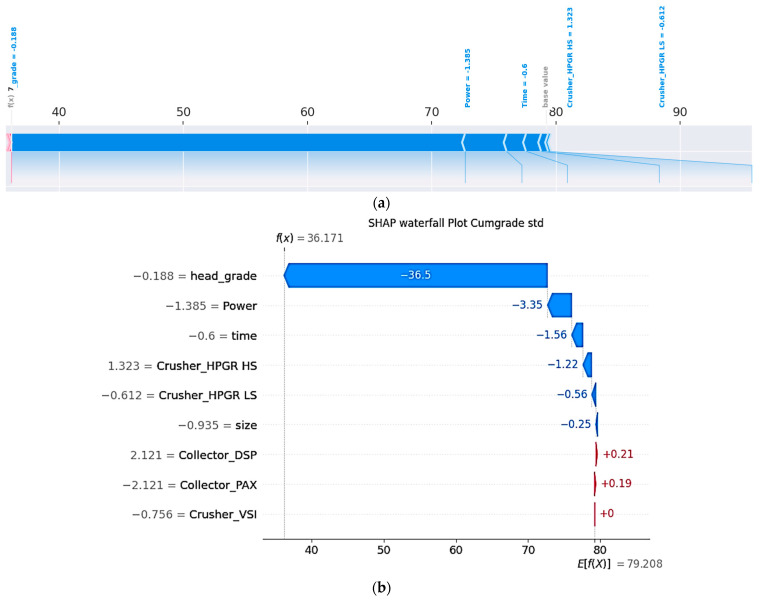
(**a**) Force plot for cumulative grade original features. (**b**) Waterfall plot for cumulative grade original features.

Cumulative Grade (Original Features)—Figure 10a,b:

Head_grade dominates with the largest positive contribution, reflecting its direct link to product quality.

Size contributes negatively (blue), indicating that finer particles reduce grade, likely due to entrainment of gangue minerals.

**Figure 11:**
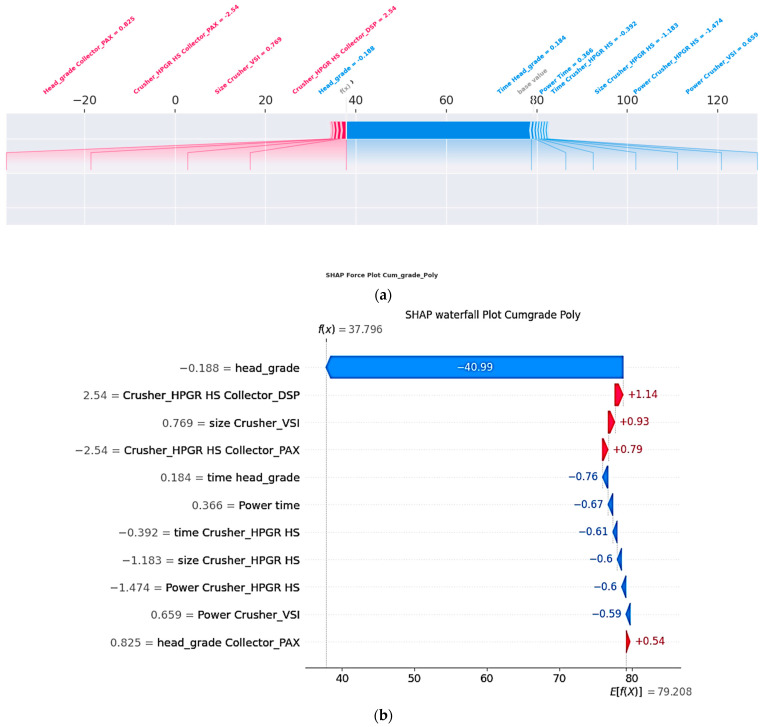
(**a**) Force plot for cumulative grade polynomial features. (**b**) Waterfall plot for cumulative grade polynomial features.

Cumulative Grade (Polynomial Features)—Figure 11a,b:

head_grade^2^ shows a negative impact, highlighting diminishing returns at very high head grades.

head_grade × time is a strong positive contributor, suggesting synergy between grade and residence time.

### 4.2. Comparative Insights

Positive Drivers: Head_grade for grade; Time for recovery; their interaction for both outcomes.

Negative Drivers: Over-grinding (time^2^) and excessive head grade (head_grade^2^) introduce inefficiencies.

Trade-offs: While longer grinding improves recovery, it can reduce grade selectivity, reinforcing the need for optimized operating windows.

Example: For a specific instance, E[f(x)] = 87.2 (baseline) and f(x) = 88.7 (prediction). The force and waterfall plots illustrate how positive contributors (e.g., time, head_grade) offset negative ones (e.g., size, excessive Power) to reach the final prediction.

## 5. SHAP Dependence Plots for Power

**Figure 12:**
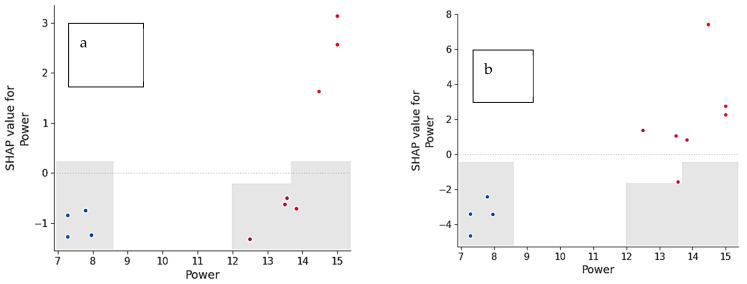
SHAP value for power vs. power plot. (**a**) Cumulative recovery; (**b**) Cumulative grade.

Figure 12a,b illustrate SHAP dependence plots for the feature Power in predicting cumulative recovery (y_1_) and cumulative grade (y_2_), respectively. These plots show how the contribution of Power (SHAP value) varies across its range (7–15 kW).

Key Observations:

Cumulative Recovery (Figure 12a):

Low Power values (7–8 kW) exhibit negative SHAP values, reducing predicted recovery.

Mid-range Power (12–13 kW) shows minimal effect (SHAP ≈ 0).

High Power (14–15 kW) produces positive SHAP values, strongly enhancing recovery.

Cumulative Grade (Figure 12b):

Higher Power (12–15 kW) contributes positively to grade, though the effect appears less pronounced than for recovery.

Unlike recovery, moderate Power levels still provide a positive contribution to grade, suggesting a different trade-off pattern.

Interpretation:

These plots confirm a non-linear relationship between Power and flotation outcomes. Higher Power favors recovery by improving liberation but may lead to diminishing returns or grade compromise. For grade, moderate-to-high Power remains beneficial, aligning with the hypothesis that controlled grinding improves selectivity.

## 6. SHAP 3D Interaction Analysis: Power × Size

**Figure 13:**
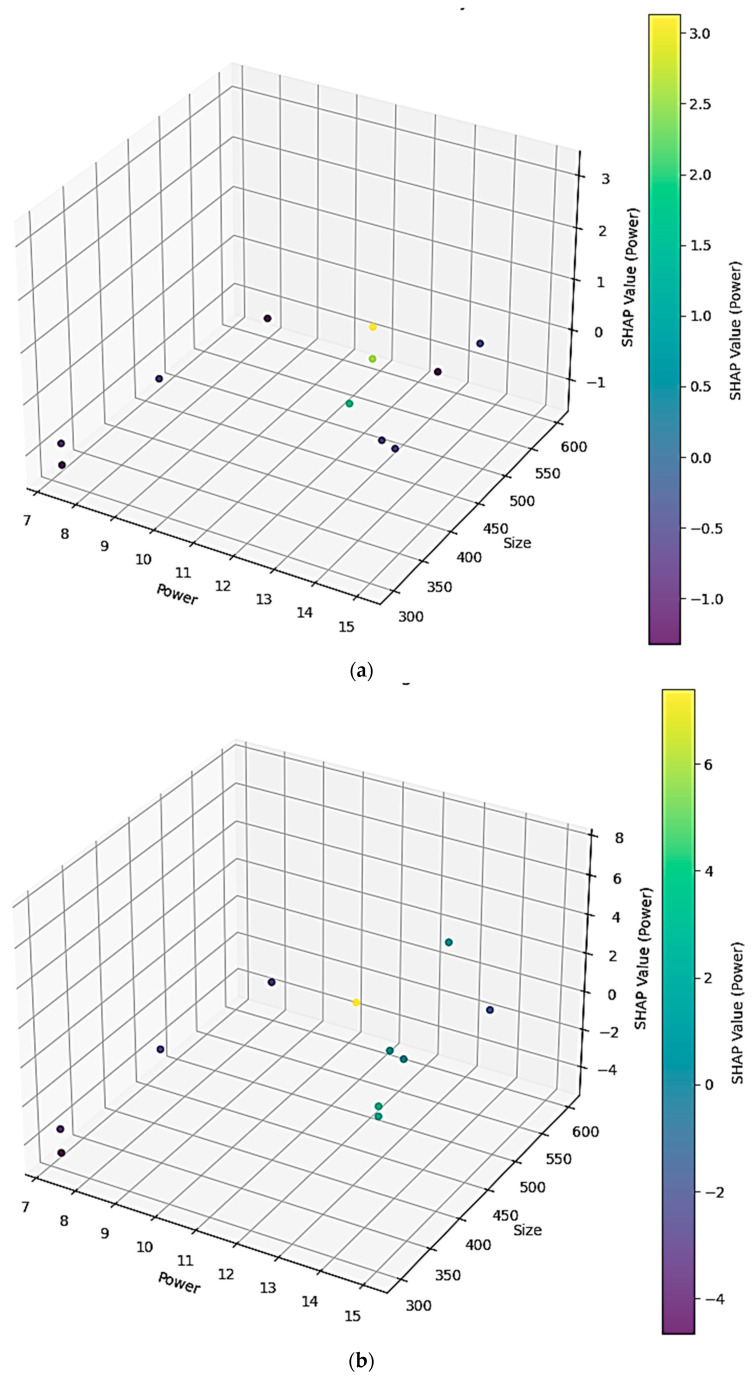
(**a**) 3D Surface Scatter Plot for “Power,” “Size,” and the SHAP value of “Power; for Cumulative recovery. (**b**) 3D Surface Scatter Plot for “Power,” “Size,” and the SHAP value of “Power; for Cumulative Grade.

Figure 13a,b illustrate the combined effect of Power and Size on the SHAP contribution of Power for cumulative recovery (y_1_) and cumulative grade (y_2_), respectively.

Key Observations:

Cumulative Recovery (Figure 13a):

At low Power (7–8 kW), SHAP values are negative across all sizes, indicating minimal contribution to recovery.

As Power increases to 10–12 kW, SHAP values become positive for larger particle sizes (>500 µm), suggesting a synergistic effect of high size and moderate Power.

Beyond 12 kW, especially when Size >450 µm, SHAP values decline again, signaling diminishing returns or potential adverse effects of excessive grinding energy.

Cumulative Grade (Figure 13b):

Optimal SHAP contributions occur when Power is between 11 and 13 kW and Size exceeds 500 µm, reflecting conditions favoring grade selectivity.

Power >13 kW does not further improve grade and may even reduce selectivity, reinforcing the trade-off between over-grinding and entrainment.

Interpretation and Process Implications:

These 3D SHAP plots reveal critical interaction thresholds rather than isolated effects: moderate Power combined with coarser Size maximizes positive impact, whereas excessive Power reduces benefits.

For practical application, operating within these identified zones can improve energy efficiency and optimize recovery-grade trade-offs, avoiding inefficiencies such as unnecessary Power consumption.

Similar interaction analysis for other variable pairs could provide additional process optimization insights.

In summary, local SHAP dependence plots (Figure 12) and 3D interaction plots (Figure 13) confirm non-linear behaviors—such as diminishing returns with head grade and trade-offs between Power and size—aligning with metallurgical theory.

In summary, SHAP analysis not only identified the most influential variables but also revealed critical interaction effects. These insights reinforce that flotation performance depends on combined operational and material characteristics rather than isolated parameters.

## 7. Sensitivity Analysis

**Figure 14:**
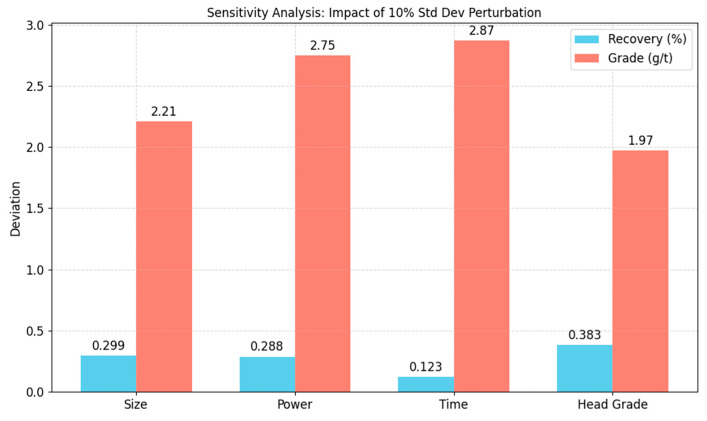
Sensitivity analysis: impact of 10% Std Dev perturbation.

A sensitivity analysis was conducted to evaluate the impact of small perturbations (10% of the standard deviation) in key numerical features—Size, Power, Time, and Head Grade—on cumulative recovery and cumulative gold grade Figure 14.

Head Grade showed the highest sensitivity for cumulative recovery, indicating its critical role in gold extraction efficiency.

Time and Power had the strongest influence on cumulative grade, suggesting that processing duration and energy input significantly affect gold concentration.

Size contributed moderately to both metrics.

These findings highlight the importance of optimizing head grade and processing conditions to enhance recovery and grade outcomes.

**Statistical Comparison of Collector Types (PAX Vs. DSP)**

A two-sample *t*-test was performed to compare cumulative recovery and grade between the two collector types:

Cumulative Recovery: t = −1.25, *p* = 0.2465 → Not statistically significant

Cumulative Grade: t = 0.50, *p* = 0.6854 → Not statistically significant

**Figure 15:**
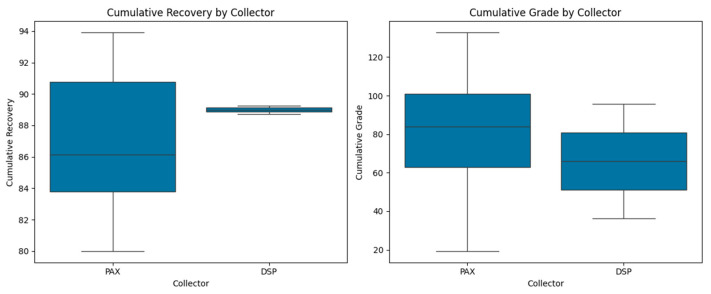
Boxplots showing visible differences for different collectors, but not statistically significant due to small sample sizes.

Both *p*-values exceed the 0.05 threshold, indicating that collector type did not have a statistically significant effect on either recovery or grade in this dataset. Boxplots (Figure 15) supported this conclusion, showing overlapping distributions.

**Categorical Analysis of Crusher Types**

**Figure 16:**
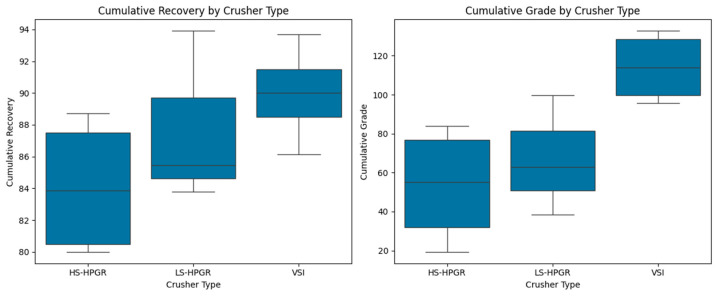
Boxplots showing how different crusher types (HS-HPGR, LS-HPGR, VSI) affect recovery and grade. VSI crushers tend to yield higher grades, while recovery varies more across types.

Crusher types were extracted and grouped into HS-HPGR, LS-HPGR, and VSI categories. Boxplots (Figure 16) revealed:

VSI crushers tended to yield higher cumulative grades.

Recovery varied across crusher types, with no single type consistently outperforming others.

This suggests that crusher selection may influence grade more than recovery, and should be considered in process design.

## 8. Limitations of the Study

While this study provides valuable insights into the variables influencing gold recovery and grade, several limitations should be acknowledged:

Dataset Size: The analysis is based on a relatively small dataset (n = 11). This limitation arises from the experimental constraints associated with preparing, crushing, and floating ore from the Ballarat gold mine under carefully controlled laboratory conditions. Such experiments are time- and resource-intensive, which restricts the number of replicates that can be realistically obtained. Despite this limitation, small datasets are not uncommon in mineral processing research, particularly when the cost and complexity of pilot-scale experimentation are considered. Previous studies have shown that explainable machine learning can still generate valid insights in small-sample contexts, especially when the focus is on interpretability rather than pure predictive performance [1,4,23]. In fact, SHAP and permutation importance have been applied effectively in other domains with limited data, where the goal was to uncover mechanistic relationships rather than to build production-ready predictive models [5,6,9].

Mineralogical Validation:

While the study discusses the potential influence of mineralogical characteristics (e.g., pyrite presence, particle shape), these inferences are based on semi-quantitative compositional data and domain knowledge. Direct validation through mineral liberation analysis, microscopy, or quantitative mineralogical tools (e.g., QEMSCAN) was not conducted. Future work should incorporate these techniques to confirm and refine the observed relationships between mineralogy and flotation performance.

Industrial Relevance: Even with limited data, the findings provide a valuable proof-of-concept demonstrating that interpretable machine learning can uncover hidden relationships between process variables. The dataset captures realistic variability in Power, head grade, time, and collector choice, making the conclusions relevant to operational practice. Small but carefully designed datasets are particularly important in mineral processing, where collecting hundreds of independent experiments is impractical. This study emphasizes the methodological contribution—showing how explainable ML can be applied in such data-constrained contexts—rather than claiming universal predictive accuracy.

Generalizability: The dataset originates from a single ore type and flotation scheme (Ballarat mine). As such, the generalizability of results to other deposits and processing conditions is limited. Future research should expand the dataset to include a broader range of ore types, additional process variables (e.g., pH, pulp density, temperature), and larger sample sizes to confirm the robustness of the identified trends.

Other Constraints: The study relies on static, pre-recorded data and does not capture temporal fluctuations or real-time operational variability. Moreover, mineralogical variables such as pyrite abundance or liberation characteristics were not directly measured, limiting the ability to confirm inferred relationships. These aspects represent important avenues for follow-up investigations.

In summary, while the dataset is small, the integration of Gradient Boosting with SHAP and permutation analysis provides statistically and industrially meaningful insights. This work demonstrates the feasibility of applying interpretable ML methods to mineral processing datasets of limited size, establishing a foundation for more comprehensive future studies.

## 9. Conclusions

This study employed advanced machine learning techniques, specifically Gradient Boosting Regressor, to analyze and interpret the key variables influencing cumulative recovery and grade in flotation processes. The integration of feature importance, permutation importance, and SHAP (Shapley Additive Explanations) analysis provided a comprehensive understanding of the factors affecting these outcomes.

Key findings include

Critical Features: Power, head grade, and time emerged as the most influential variables for both cumulative recovery and grade. These findings were consistently highlighted across feature importance, permutation importance, and SHAP analyses, reinforcing their critical roles in the flotation process.

Interaction Effects: The inclusion of polynomial features and SHAP analysis uncovered significant interaction effects between variables. For instance, interactions such as size-head grade and head grade-collector DSP were identified as critical factors for cumulative recovery, while interactions like Power-time and size-crusher VSI were important for cumulative grade. These interactions reveal the complex relationships between features that are not apparent from original features alone.

Non-linear Relationships: SHAP analysis highlighted non-linear relationships, such as diminishing returns at higher head grades and optimal ranges for Power and size. These insights are crucial for optimizing operational parameters to achieve maximum efficiency and effectiveness in the flotation process.

Local and Global Interpretability: SHAP values provided both global and local interpretability, offering detailed explanations for individual predictions and overall feature importance. This dual perspective enhances the understanding of how specific feature values influence model predictions and supports more informed decision-making.

Operational Insights: The findings from this study offer valuable operational insights for practitioners in mineral processing. By understanding the critical factors and their interactions, operators can optimize flotation conditions, adjust operational parameters in real-time, and schedule maintenance activities to prevent process disruptions. This approach ensures consistent product quality and enhances profitability in gold recovery and grade optimization.

Implications for Practice:

Optimization of Power and Head Grade: The consistent importance of Power and head grade across all analyses underscores the need for careful optimization of these parameters. Ensuring adequate energy input and high ore quality can significantly enhance recovery and grade outcomes.

Balancing Processing Time: The moderate impact of processing time, along with its interactions with other features, suggests the need for a balanced approach. Optimal grinding durations should be determined to maximize recovery without incurring excessive energy costs.

Collector Selection: The influence of collectors (PAX and DSP) on recovery highlights the importance of selecting appropriate reagents based on ore characteristics. Fine-tuning collector dosages and combinations can improve flotation performance.

Feature Interactions: The identification of significant interaction effects emphasizes the need for a holistic approach to process optimization. Understanding how different features interact can guide adjustments to operational parameters, leading to improved recovery and grade.

Future Research Directions:

Exploration of Additional Features: Future studies could explore additional features and their interactions to further enhance the understanding of factors influencing flotation processes.

Application of Other Machine Learning Models: Investigating the application of other machine learning models, such as neural networks or support vector machines, could provide complementary insights and improve predictive accuracy.

Real-time Monitoring and Control: Implementing real-time monitoring and control systems based on the insights from SHAP analysis could further optimize flotation processes and enhance operational efficiency.

## Figures and Tables

**Table 1:** Ore composition wt.% (semiquantitative) [4,10].

Phase	Weight %
Quartz	50
Muscovite	18
Fe-Dolomite/ankerite	11.5
Chlorite	7.9
Siderite	4.2
Kaolinite	3.4
Albite	3.3
Rutile	0.8
Pyrite	0.9

**Table 2:** Gold crushing and flotation data [4,10].

Crusher	Size, µm	Power (Crusher), kW	Time for Producing Required Size, s	Head Grade, g/t	Cum. Recovery, %	Cumulative Gold Grade g/t	Collector
HS-HPGR (600 µm)	600	7.97	30	2.32	80.67	74.32	PAX
LS-HPGR (600 µm)	600	13.55	41.5	1.09	85.45	38.51	PAX
VSI (600 µm)	600	5.42	28	2.70	86.15	132.92	PAX
HS-HPGR (425 µm)	425	7.80	33	2.34	87.09	83.99	PAX
LS-HPGR (425 µm)	425	13.83	95	2.12	83.80	99.77	PAX
VSI (425 µm)	425	5.52	40	2.76	90.78	126.91	PAX
HS-HPGR (300 µm)	300	7.29	36	0.84	79.98	19.15	PAX
LS-HPGR (300 µm)	300	14.48	122	2.00	93.93	62.90	PAX
VSI (300 µm)	300	5.78	50	2.85	93.69	100.85	PAX
VSI (300 µm)	300	5.78	50	2.46	89.26	95.8	DSP
HS-HPGR (300 µm)	300	7.29	36	2.02	88.72	36.17	DSP

**Table 3:** Descriptive statistics of the data [4].

Statistic	Size	Power	Time	Head Grade	Cumulative Recovery	Cumulative Grade
Count	11.00	11.00	11.00	11.00	11.00	11.00
Mean	415.91	11.66	52.82	2.14	87.23	79.21
Std	130.03	3.31	29.42	0.65	4.66	37.09
Min	300.00	7.29	30.00	0.84	79.98	19.15
25%	300.00	7.88	34.50	2.01	84.63	50.71
50%	425.00	13.50	41.50	2.32	87.09	83.99
75%	512.50	14.15	55.00	2.58	90.02	100.31
Max	600.00	15.00	122.00	2.85	93.93	132.92

**Table 4:** Description of features.

Feature Type	Feature Name	Description	Units	Role in Analysis
**Original Features**	Size	Particle size after crushing/grinding	µm	Influences liberation and flotation selectivity
	Power	Grinding energy applied to the ore	kW	Affects overgrinding and particle shape
	Time	Processing (grinding) time	min	Impacts degree of liberation
	Head Grade	Initial gold grade in feed material	g/t	Determines potential for recovery and grade
	Crusher Type	HS-HPGR, LS-HPGR, or VSI	Categorical	Affects size distribution and particle morphology
	Collector Type	PAX or DSP	Categorical	Influences gold particle flotation selectivity
**Polynomial Features**	Size^2^	Squared term to capture non-linear effects	µm^2^	Models curvature in size–response relationship
	Power^2^	Squared term for Power	(kW)^2^	Identifies diminishing returns at high Power
	Time^2^	Squared term for processing time	(min)^2^	Captures optimal grinding duration
	Head Grade^2^	Squared term for head grade	(g/t)^2^	Detects diminishing returns for grade
	Interaction Terms	E.g., Size × Head Grade, Power × Time, Head Grade × Collector	Mixed	Captures synergies and non-linear interactions

**Table 5:** Model performance summary (MSE).

Model	Target	Features	MSE
Gradient Boosting	Cum recovery	Original	32.74
Gradient Boosting	Cum recovery	Polynomial	30.35
Random Forest	Cum recovery	Original	23.72
Random Forest	Cum recovery	Polynomial	23.30
Gradient Boosting	Cumulative grade	Original	841.17
Gradient Boosting	Cumulative grade	Polynomial	633.67
Random Forest	Cumulative grade	Original	668.19
Random Forest	Cumulative grade	Polynomial	824.62

**Table 6:** Comparison of force plots and waterfall plots.

Aspect	Force Plot	Waterfall Plot
Visualization Style	Compact, uses arrows for feature contributions	Step-by-step, sequential adjustment
Base Value (E[f(x)])	Shown at the ~center as the starting point	Shown at the top as the starting point
Final Prediction (f(x))	Displayed on the far right	Displayed at the bottom
Best Use Case	Comparing feature impacts across instances	Detailed breakdown for a single instance

## Data Availability

The original contributions presented in this study are included in the article in Table 2. Further inquiries can be directed to the corresponding author.

## References

[1] ReferencesChelgani S.C., Homafar A., Nasiri H., Laksar M.R. (2024). CatBoost-SHAP for modeling industrial operational flotation variables–A. Miner. Eng..

[2] Gholamreza B., Hamid K., Reza G.M., Mostafa M., Ahmad H. (2023). Metallurgical evaluation of copper ore flotation performance in the presence of Rhamnolipid biosurfactant produced from Pseudomonas aeruginosa. Part 1: Copper-bearing minerals. Physicochem. Probl. Miner. Process..

[3] Zhang Z., Zhang T., Li J. (2023). Unbiased Gradient Boosting Decision Tree with Unbiased Feature Importance. arXiv.

[4] Devasahayam S. (2025). Advancing Flotation Process Modeling: Bayesian vs. Sklearn Approaches for Gold Grade Prediction. Minerals.

[5] Chamma A., Engemann D.A., Thirion B. (2023). Statistically Valid Variable Importance Assessment through Conditional Permutations. arXiv.

[6] Fumagalli F., Muschalik M., Hüllermeier E., Hammer B. (2023). Incremental Permutation Feature Importance (iPFI): Towards Online Explanations on Data Streams. Mach. Learn..

[7] Zhao C., Liu J., Parilina E. (2025). ShapG: New Feature Importance Method Based on the Shapley Value. Eng. Appl. Artif. Intell..

[8] Letoffe O., Huang X., Asher N., Marques-Silva J. (2024). From SHAP Scores to Feature Importance Scores. arXiv.

[9] Wang H., Liang Q., Hancock J.T., Khoshgoftaar T.M. (2024). Feature selection strategies: A comparative analysis of SHAP-value and importance-based methods. J. Big Data.

[10] Thatipamula S., Devasahayam S. (2025). Energy-Efficient Gold Flotation via Coarse Particle Generation Using VSI and HPGR Comminution. Materials.

[11] Thatipamula S., Devasahayam S. (2024). Study of Coarse and Fine Gold Flotation on the Products from Vertical Shaft Impactor and High-Pressure Grinding Roll Crushers. https://ssrn.com/abstract=4727563.

[12] Koehrsen W. (2018). Visualizing Data with Pairs Plots in Python. https://towardsdatascience.com/visualizing-data-with-pair-plots-in-python-f228cf529166.

[13] Fu H., Wang Z., Nichani E., Lee J.D. (2024). Learning Hierarchical Polynomials of Multiple Nonlinear Features with Three-Layer Networks. arXiv.

[14] Choudhury A. (2021). AdaBoost Vs Gradient Boosting: A Comparison of Leading Boosting Algorithms. https://analyticsindiamag.com/adaboost-vs-gradient-boosting-a-comparison-of-leading-boosting-algorithms/#:~:text=AdaBoost%20is%20the%20first%20designed,Boosting%20more%20flexible%20than%20AdaBoost.

[15] Aaltonen A., Izart C., Lyyra M., Lang A., Saari E., Dahl O. (2023). Simulating the Impact of Ore and Water Quality on Flotation Recovery during the Life of a Mine. Minerals.

[16] Dhar P., Thornhill M., Kota H.R. (2019). Investigation of Copper Recovery from a New Copper Deposit (Nussir) in Northern. Miner. Process. Extr. Metall. Rev..

[17] Mohammadi V.S., Noaparast M., Aslani S. (2022). The effect of high voltage electrical pulses on iron ore comminution to improve desulfurization flotation recovery. Physicochem. Probl. Miner. Process..

[18] Sirkeci A., Gul A., Bulut G., Özer M., Güven O., Perek K. (2018). Effect of crushing type on the efficiency of flowing film separation. Physicochem. Probl. Miner. Process..

[19] Guven O., Serdengecti M.T., Tunc B., Ozdemir O., Karaagaclioglu I.E., Çelik M.S. (2020). Effect of particle shape properties on selective separation of chromite from serpentine by flotation. Physicochem. Probl. Miner. Process..

[20] 911Metallurgist (2016). Size by Size Particle Size and Flotation Recovery.

[21] Allan G., Woodcock J. (2001). A review of the flotation of native gold and electrum. Miner. Eng..

[22] Özçelik S., Ekmekçi Z. (2024). Surface Chemistry and Flotation of Gold-Bearing Pyrite. Minerals.

[23] Devasahayam S., Thella J.S., Mohanty M.K. (2025). Predicting Methane Dry Reforming Performance via Multi-Output Machine Learning: A Comparative Study of Regression Models. Energies.

